# Rupioid Syphilis Beyond Immunosuppression: A Systematic Review of Cases in HIV-Negative Patients

**DOI:** 10.7759/cureus.116910

**Published:** 2026-09-25

**Authors:** Erica K Rankin, Chad Gentry, Alexandra Daily, Julianna Foss, Aishwi Patel, Dalton Shepherd, Kelsey Voss, Julia Juriga, Kimberly A Kluglein, Suzanne I Riskin

**Affiliations:** 1 Medicine, Department of Foundational Sciences, Nova Southeastern University Dr. Kiran C. Patel College of Osteopathic Medicine, Clearwater, USA; 2 Dermatology, Hospital Corporation of America (HCA) Healthcare Florida Largo Hospital/University of South Florida (USF) Morsani College of Medicine, Largo, USA

**Keywords:** immunocompetent, jarisch-herxheimer reaction, lues maligna, malignant syphilis, penicillin g benzathine, rapid plasma reagin, rupioid syphilis, syphilis, treponema pallidum, venereal disease research laboratory

## Abstract

Rupioid syphilis (malignant syphilis or lues maligna) is a severe ulcero-nodular manifestation of infection originating from *Treponema pallidum*. It is typically associated with immunosuppressed states, notably those derived from HIV co-infection. This systematic review aims to highlight the rare instances in which this multi-faceted disease affects those with HIV seronegative immune status. A review of cases was conducted using PubMed, Embase, and Web of Science databases with Boolean operators utilizing all variants of “malignant syphilis” or “lues maligna” or “rupioid syphilis” combined with “immunocompetent.” Relevant studies included those from inception through August 2026. After removal of duplicates and screening, a total of 20 cases were selected based on inclusion criteria. The results revealed consistent clinical features, including systemic symptoms and ulcerative-necrotic lesions in 20 patients lacking underlying HIV immunosuppression. These findings underscore the importance of maintaining high clinical suspicion for syphilis in patients presenting with necrotic papuloulcerative lesions and systemic manifestations, regardless of HIV status, to ensure timely diagnosis and management.

## Introduction and background

*Treponema pallidum* is a Gram-negative spirochete responsible for syphilis, a bacterial infection with life-threatening consequences of systemic infection if left untreated [1]. Inoculation begins when the bacterium pierces through mucous membranes or microscopic dermal abrasions, with initial infection sites involving the vaginal, anal, or oral passages [2]. Transmission can occur both sexually and vertically, with a recent rise in incidence affecting high-income countries [3]. In its primary stage, syphilis presents as a single, painless chancre most commonly affecting the genital region. When individuals with signs of initial inoculation are left untreated, syphilis infection can progress through secondary, latent, and tertiary stages of syphilis, with the potential of the bacterium to invade multiple organ systems [2]. 

Individuals with clinical exam findings raising suspicion for a syphilitic infection, such as a painless ulcer or maculopapular rash affecting the palms and soles, will undergo diagnostic testing for confirmation. Treponemal and nontreponemal serology remains the diagnostic method of choice, with providers most commonly utilizing fluorescent antibody-absorption IgG/IgM (FTA-ABS), the Venereal Disease Research Laboratory (VDRL) test, and the rapid plasma reagin (RPR) test [4]. The VDRL and RPR are non-treponemal tests used to monitor disease activity [4]. Direct detection using dark-field microscopy, silver stain histochemistry, immunohistochemistry, and PCR are additional methods that providers may utilize in patients presenting with clinically suspected syphilis with negative or equivocal serology [4]. A single intramuscular (IM) dose of 2.4 million units (MU) penicillin G benzathine is the mainstay treatment of syphilis, with latent presentations sometimes requiring additional injections of 2.4 MU penicillin G benzathine one week apart [3].

Treatment is considered successful when nontreponemal testing titers show a minimum four-fold reduction [4]. Spirochetal lysis following initiation of antibiotic therapy can result in a transient inflammatory response with systemic symptoms, described as a Jarisch-Herxheimer reaction (JHR) [5]. It occurs in almost 30% of patients who receive treatment and more commonly occurs among those with secondary syphilis [3]. This reaction is self-limiting, with resolution of symptoms within 24 hours [3]. 

Rupioid syphilis (also known as lues maligna, malignant syphilis) is a rare form of secondary syphilis. Various theories regarding the pathogenesis of rupioid syphilis have been reported, with development influenced by an abnormally elevated immune response, interaction with HIV, or increased virulence of *T. pallidum* [6-9]. Rupioid syphilis differentiates itself from secondary and tertiary syphilis by its clinical presentation. Coined as rupioid syphilis due to its unique oyster-shell-like appearance, these lesions present as well-demarcated, round to oval ulcerative nodules with thick, dark, adherent crusts in a symmetrical, generalized distribution that typically spares the palms and soles [10]. The lesions often follow a prodromal phase of fever, myalgia, arthralgia, anorexia, and headache that is more abrupt in onset compared to other syphilitic phases [6]. Diagnosis is based upon the presence of characteristic clinical features, histopathology showing sparse or absent spirochetes in the tissue, high serologic titers, a JHR, and rapid improvement following appropriate treatment [6]. Histology demonstrates lymphocytic predominance and plasma cells with dermal infiltrate [11]. A lack of specificity lends itself to a diagnosis of exclusion [11]. Systemic involvement can occur with malignant syphilis, as reports have documented elevated liver enzymes, ocular inflammation, and neurosyphilis [11]. Treatment for malignant syphilis remains three doses of 2.4 MU penicillin G benzathine given one week apart, with expected complete resolution [11].

Rupioid syphilis most commonly affects immunosuppressed populations, particularly HIV-positive patients. While rare, reports of rupioid syphilis have been documented among HIV-negative individuals. Between 2014 and 2018, 27% of reported cases occurred in HIV-negative individuals [12]. Half of these cases had comorbidities such as hepatitis, psoriasis, diabetes mellitus, or substance abuse [12]. 

With the increasing incidence of syphilis, providers should be mindful of the uncommon cutaneous manifestations and potential to present in HIV-negative hosts [3]. Adequate suspicion for this infrequent infection in HIV-negative patients can advance diagnosis and treatment time, helping to prevent the spread and potential health consequences. Here, we identify reports of rupioid syphilis in HIV-negative hosts. The objective of this systematic review is to provide an updated, comprehensive review of the literature as well as identify potential clinical patterns or comorbidities that can contribute to the development of this disease in HIV-negative patients.

## Review

Methods

Literature Search

An electronic search of three databases, PubMed, Embase, and Web of Science, was conducted on August 21, 2026. Key terms searched included "rupioid syphilis," "malignant syphilis," and "lues maligna," using Boolean operators “OR” and "AND." Our exact search strategy and date of search have been summarized in Appendix A.

Inclusion and Exclusion Criteria

Certain criteria were selected to best adhere to the goal of our review. Included articles consisted of those that were peer-reviewed, written in English, published from inception through August 2026, and described cases of malignant syphilis in serologically proven HIV-negative patients. Excluded articles included non-English language articles and those that did not pertain to malignant syphilis or described malignant syphilis in HIV-positive patients.

Study Identification, Selection, and Data Extraction

Selection of articles was conducted in three stages. Organization of data was supported by Rayyan (Rayyan Systems Inc., Cambridge, MA, USA), a platform used to screen and organize for reviews. First, titles and abstracts were screened for relevance. Next, a full-text review of selected articles allowed for final selection. Lastly, data were extracted and organized. Our initial search yielded a total of 157 articles. Of the initial articles, 36 duplicates were found. Removal of duplicate articles resulted in a total of 121 articles for initial review. Titles and abstracts were reviewed by two reviewers for inclusion, after which the full text of 29 articles was reviewed. A total of 20 full-text articles were included in this case series. Conflicts were resolved through discussion and a third reviewer if discussion was unsuccessful. The study selection process is shown using a PRISMA-ScR flow diagram (Figure 1) [13]. A summary of the studies is featured in Appendix B.

**Figure 1:**
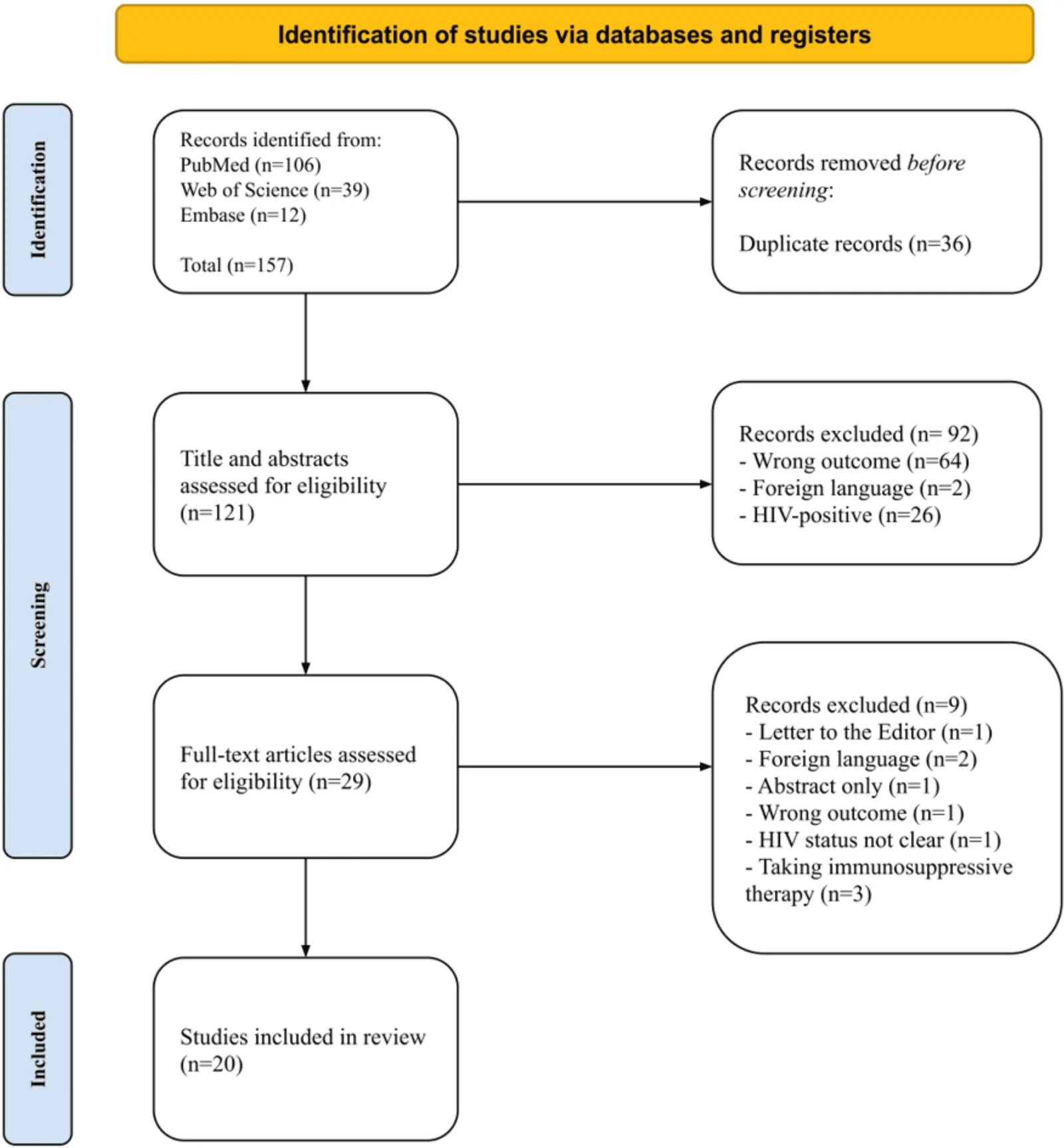
PRISMA-ScR flow diagram of the search and selection process PRISMA-ScR: Preferred Reporting Items for Systematic reviews and Meta-Analyses extension for Scoping Reviews [13]

Risk of Bias Assessment

Eligible articles underwent quality assessment with the Joanna Briggs Institute (JBI) Critical Appraisal Tools for Case Reports Studies [14]. Reports categorized as low (>70%) were included in our final review, while moderate (50% to 70%) and high (<50%) risk of bias based on appraisal scores were excluded (Table 1).

**Table 1:** Risk of bias domains using the JBI critical appraisal tools for case reports studies D1: Were the patient’s demographic characteristics clearly described? D2: Was the patient’s history clearly described and presented as a timeline? D3: Was the current clinical condition of the patient on presentation clearly described? D4: Were diagnostic tests or assessment methods and the results clearly described? D5: Was the intervention(s) or treatment procedure(s) clearly described? D6: Was the post-intervention clinical condition clearly described? D7: Were adverse events (harms) or unanticipated events identified and described? D8: Does the case report provide takeaway lessons? ★ No; ✦ Unclear; ⬤ Yes; ✹ Not applicable JBI: Joanna Briggs Institute [14]

Study ID	D1	D2	D3	D4	D5	D6	D7	D8	Overall
Xará et al., 2024 [15]	★	⬤	⬤	⬤	⬤	⬤	⬤	⬤	⬤
Chen et al., 2022 [16]	⬤	⬤	⬤	⬤	⬤	⬤	⬤	⬤	⬤
Barit et al., 2023 [17]	⬤	⬤	⬤	⬤	⬤	⬤	⬤	⬤	⬤
Margulies et al., 2022 [18]	⬤	⬤	⬤	⬤	⬤	⬤	⬤	⬤	⬤
Sá Lopes et al., 2023 [19]	⬤	⬤	⬤	⬤	⬤	⬤	⬤	⬤	⬤
Requena et al., 2014 [8]	⬤	⬤	⬤	⬤	⬤	⬤	⬤	⬤	⬤
Dueñaz-Díaz et al., 2025 [20]	⬤	⬤	⬤	⬤	⬤	⬤	⬤	⬤	⬤
Al-Abdulla et al., 2025 [21]	⬤	⬤	⬤	⬤	⬤	★	⬤	⬤	⬤
Rao et al., 2017 [22]	⬤	⬤	⬤	⬤	⬤	★	⬤	⬤	⬤
Demirbaş et al., 2022 [23]	⬤	⬤	⬤	⬤	⬤	★	⬤	⬤	⬤
Bayramgüler et al., 2005 [24]	⬤	⬤	⬤	⬤	⬤	⬤	⬤	⬤	⬤
Watson et al., 2004 [25]	⬤	⬤	⬤	⬤	★	⬤	⬤	⬤	⬤
Dimnik et al., 2022 [26]	⬤	⬤	⬤	⬤	⬤	⬤	⬤	⬤	⬤
Rockwood & Nwokolo, 2018 [27]	⬤	⬤	⬤	⬤	⬤	⬤	⬤	⬤	⬤
Bertrand et al., 2012 [10]	⬤	⬤	⬤	⬤	⬤	⬤	⬤	⬤	⬤
Nguyen et al., 2022 [28]	⬤	⬤	⬤	⬤	⬤	★	⬤	⬤	⬤
de Unamuno Bustos et al., 2014 [29]	⬤	⬤	⬤	⬤	⬤	⬤	⬤	⬤	⬤
Ortigosa et al., 2016 [9]	⬤	⬤	⬤	⬤	⬤	⬤	⬤	⬤	⬤
Alves et al., 2015 [30]	⬤	⬤	⬤	⬤	⬤	⬤	⬤	⬤	⬤
Mazzola et al., 2025 [31]	⬤	⬤	⬤	⬤	⬤	⬤	⬤	⬤	⬤

Results

Our review highlights 20 cases of rupioid syphilis in serology-proven HIV-negative patients, an unusual population to be affected by this rare latent disease (Appendix B). Demographics revealed patients aged 20 to 60 years old and equal sex distribution. Some patients have a past medical history that could potentially contribute to an immunocompromised state (psoriasis: n=2; HBV: n=2; chronic alcoholism: n=1; diabetes mellitus: n=1; homelessness, polysubstance use disorder, and diabetes mellitus: n=1) [8,9,23,24,28,29,31]. Onset of lesions ranged from 15 days to three months before patients sought medical assessment. All patients presented with ulcerated or crusted papulonodular eruptions that were spread throughout the body, most commonly involving the face, trunk, and extremities. A prodrome of fever, malaise, and lymphadenopathy preceded eruption. Histopathologic findings were consistent with plasma cell-rich granulomatous dermatitis and vasculitis. Ocular, neurologic, and systemic involvement in several patients underscored the disseminated potential of *T. pallidum* even in individuals without immune compromise. High nontreponemal titers (commonly ≥ 1:64) confirmed active secondary syphilis. Penicillin G benzathine 2.4 MU weekly for three weeks remained the treatment of choice, utilized in 50% of cases [8,9,15,16,19,21,23,24,29,30]. Other treatment options included amoxicillin, IV penicillin, doxycycline, and ceftriaxone [10,17,18,26,27,31]. A JHR occurred in only 20% of cases, deviating from the traditional requirement for rupioid syphilis diagnosis [8,10,15,16].

Discussion

Rupioid syphilis is a rare, ulceronecrotic subtype of secondary syphilis [28]. While the pathogenesis of rupioid syphilis is largely unknown, it is suggested that HIV immunosuppression allows increased virulence of* T. pallidum*, even in patients with relatively preserved immune function or higher CD4 strata [3,11]. Diagnostic criteria of this unique variant encompass four key findings: characteristic clinical and histopathologic morphology, high-titer treponemal serology, a JHR, and resolution following treatment with benzathine penicillin G [11]. Historically associated with severe immunosuppression, most notably HIV infection, these findings expand the clinical paradigm by highlighting other potential modifiers of host immunity, including metabolic, chronic inflammatory, hepatic, psychiatric, and iatrogenic factors.

The clinical trends found are supported by a recent review of the literature, including many of the same studies we referenced [31]. However, the authors reviewed malignant syphilis in immunocompetent hosts, including one patient who lacked HIV-negative serology [31]. Interestingly, this review included two reports of rupioid syphilis in non-immunosuppressed patients that did not populate in our literature search [31]. This review excluded reports of HIV-negative individuals with other conditions that may have contributed to a weakened immune system. Collectively, our findings suggest that rupioid syphilis should not be excluded based on HIV-negative serostatus. Subclinical immunologic or metabolic disturbances may predispose otherwise healthy individuals to severe variants of secondary syphilis. Misdiagnosis remains common, delaying definitive therapy.

Despite having HIV-negative serostatus, a past medical history could have contributed to the development of rupioid syphilis. In diabetes mellitus, hyperglycemia can impair neutrophil chemotaxis and opsonization as well as suppress type I interferon responses, reducing the body’s defense system against pathogens [32,33]. Interestingly, in one of the studies, the author made a point of disclosing that the patient’s hemoglobin A1c was within normal limits, despite a lack of diabetic medication [28]. Chronic alcoholism and polysubstance use disorder are well-known to carry elevated infectious disease risk, as drugs of abuse carry immunosuppressive effects [34]. Homelessness can magnify this through increased risks of exposure, malnutrition, and limited access to healthcare. Patients with psoriasis can exhibit increased susceptibility to infection, though usually related to systemic immunosuppression caused by agents treating the disease [35]. However, in another study, two patients with a history of psoriasis were not using systemic therapies, and only one patient was being treated with a topical regimen [15,31]. Nonetheless, over half of the patients highlighted in this review are HIV-negative with intact immune systems.

Limitations should be considered when interpreting these findings. Publication bias may be present, as cases with unusual presentations, severe disease, or favorable outcomes are more likely to be reported. This review was not registered on PROSPERO (International Prospective Register of Systematic Reviews); however, we attempted to avoid reporting bias by assessing each article with the JBI Critical Appraisal Tools for Case Reports Studies [14]. Many reports lacked complete demographic, histopathologic, or immunologic data, and documentation of comorbidities, serologic follow-up, or treatment response was inconsistent. Several patients were lost to follow-up, preventing confirmation of long-term outcomes or relapse rates. The absence of standardized histopathologic or microbiological confirmation across all cases introduces an additional limitation, potentially impacting diagnostic certainty.

## Conclusions

Rupioid syphilis, while classically described in immunocompromised hosts, is recognized among immunocompetent individuals. The reviewed cases demonstrate the impact of maintaining high clinical suspicion for rupioid syphilis in patients presenting with ulcer-necrotic crusted lesions and systemic symptoms, even in the absence of known immunodeficiency. Early recognition through serologic and histopathologic confirmation, timely administration of penicillin G benzathine, and vigilant follow-up can aid in preventing progression and transmission.

## Appendices

Appendix A 

**Table 2:** Summary of complete search strategy used across PubMed, EMBASE, and Web of Science conducted on August 21, 2026

Database	Search no.	Keywords	Yield of articles
PubMed	#1	“Malignant Syphilis” OR “rupioid syphilis” OR “lues maligna”	1,905
	#2	“Syphilis” OR “secondary syphilis” OR “secondary leus” OR “Treponema pallidum” OR “T. pallidum” OR “syphilis infection” OR “infection by Treponema pallidum”	53,852
	#3	“immunocompetent” OR “immune intact” OR “HIV negative” OR “human immunodeficiency virus negative”	105,868
	#4	#1 AND #2 AND #3	106
Embase	#1	“Malignant Syphilis” OR “rupioid syphilis” OR “lues maligna”	1,044
	#2	“Syphilis” OR “secondary syphilis” OR “secondary leus” OR “Treponema pallidum” OR “T. pallidum” OR “syphilis infection” OR “infection by Treponema pallidum”	3,865
	#3	“immunocompetent” OR “immune intact” OR “HIV negative” OR “human immunodeficiency virus negative”	61,239
	#4	#1 AND #2 AND #3	12
Web of Science	#1	“Malignant Syphilis” OR “rupioid syphilis” OR “lues maligna”	313
	#2	“Syphilis” OR “secondary syphilis” OR “secondary leus” OR “Treponema pallidum” OR “T. pallidum” OR “syphilis infection” OR “infection by Treponema pallidum”	26,001
	#3	“immunocompetent” OR “immune intact” OR “HIV negative” OR “human immunodeficiency virus negative”	84,980
	#4	#1 AND #2 AND #3	39

Appendix B 

**Table 3:** Summary of reported cases of rupioid (malignant) syphilis in immunocompetent individuals ALT: Alanine aminotransferase, EBV: Epstein-Barr virus, FTA-ABS: Fluorescent treponemal antibody test, GGT: Gamma glutamyl transferase, HAV: Hepatitis A virus, HBV: Hepatitis B virus, HCV: Hepatitis C virus, IHC: Immunohistochemistry, IM: Intramuscular, JHR: Jarisch-Herxheimer reaction, PIH: Post-inflammatory hyperpigmentation, RPR: Rapid plasma reagin, VDRL: Venereal Disease Research Laboratory, TPHA: Treponema pallidum hemagglutination assay, TPPA: Treponema pallidum particle agglutination assay, TRUST: Toluidine red unheated serum test

Study characteristics	Patient demographics	Lesions and onset	Serology, immunostaining, microorganism staining	Treatment	Post-treatment course
Xará et al., 2024 [14]	20F, negative HIV, hepatitis, toxoplasma, and Epstein-Barr virus (EBV)	Neck, trunk, arms; onset rapid	Treponemal+, non-treponemal+	Penicillin G benzathine 2.4 MU IM weekly ×3	JHR, then resolution in one month with residual scarring
Chen et al., 2022 [15]	26F, negative HIV, HBV, HCV	Erythematous scaly plaques on forehead, bilateral eyelids, lower jaw, left axilla, finger web spaces, anogenital areas with ulcers in the plaques affecting her axilla, onset 15 days	Toludine red unheated serum test toluidine red unheated serum test (TRUST) titre of 1:128 and TPPA+	Penicillin G benzathine 2.4 MU IM weekly ×3	JHR, regression of lesions after three weeks of treatment with post-inflammatory hyperpigmentation (PIH). TRUST titre decreased to 1:16.
Barit et al., 2023 [16]	26M, negative HIV and hepatitis	Lower limbs, onset two months	RPR+, *T. pallidum* hemagglutination assay (TPHA)+, plasma cell infiltrate, fibrinoid degeneration, immunohistochemistry (IHC) positive for *T. pallidum*	Amoxicillin 2.25 g/day + Probenecid 750 mg × four weeks	Gradual healing w/ hyperpigmentation, RPR decreased four-fold
Margulies et al., 2022 [17]	28F, negative HIV	Face, eyelids (ocular), ankles, shins, onset six weeks	RPR 1:128, IgG+, lichenoid granulomatous dermatitis with plasma cells	IV penicillin G ×14 days + sub-Tenon triamcinolone	Full recovery including cutaneous and ocular resolution
Sá Lopes et al., 2023 [18]	29F, negative HIV, HBV, and HCV	Scalp, face, neck, trunk, palms/soles, perianal, onset six weeks	RPR 1:128, fluorescent treponemal antibody test (FTA-ABS)+, subcorneal pustules, granulomas, lymphoplasmacytic infiltrate	Penicillin G benzathine 2.4 MU IM weekly ×3	Rapid healing in one month, RPR decreased to 1:4
Requena et al., 2014 [8]	29F, negative HIV, HBV, and HCV, psoriasis	Face, thorax, upper limbs, palms, onset two weeks	VDRL 1:256, lichenoid infiltrate rich in plasma cells, anti-*T. pallidum* IHC+	Penicillin G benzathine 2.4 MU IM weekly ×3	JHR, VDRL decreased 1:16 at three months, negative when measured at nine months
Dueñaz-Díaz et al., 2025 [19]	30M, negative HIV, HBV	Scalp, face, trunk, extremities, palms/soles, onset two months	VDRL 1:64 to 1:256, FTA-ABS+, epidermal hyperplasia, vasculitis, plasma cell infiltrate	Penicillin G benzathine 2.4 MU IM ×1	Rapid and complete resolution; VDRL decreased to 1:4 measured at six months
Al-Abdulla et al., 2025 [20]	34M, negative HIV antigen and antibody combo and negative HIV viral load	Erythematous nodules with collarette scales and some central ulceration on the face, trunk, and limbs, onset three weeks	Positive *T. pallidum* antibody, RPR 1:128, dense perivascular lymphohistiocytic infiltration with focal neutrophils and dense plasma cell infiltrate	Penicillin G benzathine 2.4 MU IM ×3	Complete resolution
Rao et al., 2017 [21]	35M, negative HIV	Face, arms, trunk, onset one month	VDRL 1:32, TPHA 1:160	Penicillin G benzathine 2.4 MU IM ×1	Complete resolution
Demirbaş et al., 2022 [22]	35M, negative HIV, positive HBV (undetectable)	Abdomen, palms/soles, genitalia, onset three weeks	VDRL+, TPHA+, lymphohistiocytic infiltrate with plasma cells	Penicillin G benzathine 2.4 MU IM weekly ×3 + topical care	Marked improvement in four days, lost to follow-up
Bayramgüler et al., 2005 [23]	37M, negative HIV, hepatitis A virus (HAV), HBV, HCV, chronic alcoholism	Erythematous papules, pustules and noduloulcerative lesions on face, trunk and extremities, onset 10 weeks	RPR+, VDRL 1:16, TPHA 1:80, epidermal ulceration and irregular acanthosis with diffuse plasma cell-rich infiltration throughout dermis on biopsy	Penicillin G benzathine 2.4 MU IM weekly ×3 + topical care	Initial improvement three days after first penicillin injection with complete clearance in two weeks. At three months, VDRL was reactive at 1/256, and TPHA was reactive at 1:32 at six months.
Watson et al., 2004 [24]	41F, negative HIV, elevated liver function (alanine transaminase (ALT): 195 IU/L; gamma glutamyl transferase (GGT): 114 IU/L)	Erythematous papules on chest and back that spread to face and scalp, onset six weeks	Negative RPR, but TPHA+, VDRL 1:32,	Penicillin G benzathine 2.4 MU IM	Almost complete resolution at four weeks, lost to follow-up
Dimnik et al., 2022 [25]	41F, negative HIV, HBV, and HCV	Scalp, face, neck, oral/laryngeal mucosa, trunk, arms; onset three months	RPR 1:128, TPPA >1:20,480, CSF TPPA 1:320, dense atypical T-cell infiltrates, plasma cells, granulomatous dermatitis, Warthin-Starry positive for spirochetes	IV benzylpenicillin 18 MU/day ×14 days + corticosteroids	Afebrile, full resolution, RPR decreased to 1:4 measured at 10 months
Rockwood & Nwokolo, 2018 [26]	41M, negative HIV, penicillin allergy	Face, trunk, upper limbs, testis, lungs, bone, onset two weeks	RPR 1:256, *T. pallidum* particle agglutination assay (TPPA)+, granulomatous orchitis, chronic dermatitis, IHC positive for *T. pallidum*	Doxycycline 200 mg twice daily ×4 weeks	Complete systemic and cutaneous resolution
Bertrand et al., 2012 [10]	43F, negative HIV	Scalp, face, trunk, extremities, onset two months	RPR 1:128, lymphohistiocytic infiltrate with plasma cells, immunochemistry negative for *T. pallidum*	IV penicillin G ×14 days	JHR, rapid recovery, RPR decreased 1:64 measured at four weeks
Nguyen et al., 2022 [27]	43F, negative HIV, positive for type 2 diabetes mellitus (T2DM), schizophrenia, homeless, polysubstance use disorder	Diffuse erythematous nodules and plaques with rolled borders, ulceration and crusting, onset unknown	RPR 1:128, treponemal immunoglobulin G antibody test+, epidermal acanthosis with diffuse dermal infiltrate comprised of plasma cells on biopsy	Penicillin G benzathine 2.4 MU IM	RPR decreased to 1:32 at two weeks, rash improvement is unknown, lost to follow-up
de Unamuno Bustos et al., 2014 [28]	46M, negative HIV and HCV, positive HBV	Scalp, ocular, trunk, extremities, genitals, onset two months	VDRL 1:32, FTA-ABS+, lymphohistiocytic infiltrate with plasma cells, no spirochetes visualized	Penicillin G benzathine 2.4 MU IM weekly ×3	Lesions and ocular symptoms resolved, RPR decreased fourfold
Ortigosa et al., 2016 [9]	53F, negative HIV, T2DM	Face, trunk, extremities, palms/soles, onset 20 days	VDRL 1:8, FTA-ABS+, interface dermatitis, granulomas, vasculitis	Penicillin G benzathine 2.4 MU IM ×3 + prednisone 60 mg/day	Full resolution, no relapse
Alves et al., 2015 [29]	57M, negative HIV, HBV, and HCV	Head, neck, trunk, limbs, onset four months	RPR >128, TPHA 5120, plasma cell-rich dermal infiltrate, granulomas	Penicillin G benzathine 2.4 MU IM weekly ×3	Four-fold RPR decrease measured at one year
Mazzola et al., 2025 [30]	60M, negative HIV, HBV, HCV; hypercholesterolemia, psoriasis	Multiple papular lesions with one ulcerated lesion on trunk and back, onset three months	RPR 1:128, treponemal antibodies > 70 index	Ceftriaxone 2 g/day IV x 5 days followed by penicillin G benzathine 2.4 MU IM weekly ×1	Rapid clinical improvement, normalization of inflammatory markers, and decrease in RPR to 1:32 at the sixth-month follow-up
